# Establishing Age-Adjusted Reference Ranges for Iris-Related Parameters in Open Angle Eyes with Anterior Segment Optical Coherence Tomography

**DOI:** 10.1371/journal.pone.0147760

**Published:** 2016-01-27

**Authors:** Jeffrey R. Peterson, Lauren S. Blieden, Alice Z. Chuang, Laura A. Baker, Mohammed Rigi, Robert M. Feldman, Nicholas P. Bell

**Affiliations:** 1 Ruiz Department of Ophthalmology and Visual Science, The University of Texas Health Science Center at Houston (UTHealth) McGovern Medical School, Houston, Texas, United States of America; 2 Robert Cizik Eye Clinic, Houston, Texas, United States of America; University of Iowa, UNITED STATES

## Abstract

**Purpose:**

Define criteria for iris-related parameters in an adult open angle population as measured with swept source Fourier domain anterior segment optical coherence tomography (ASOCT).

**Methods:**

Ninety-eight eyes of 98 participants with open angles were included and stratified into 5 age groups (18–35, 36–45, 46–55, 56–65, and 66–79 years). ASOCT scans with 3D mode angle analysis were taken with the CASIA SS-1000 (Tomey Corporation, Nagoya, Japan) and analyzed using the Anterior Chamber Analysis and Interpretation software. Anterior iris surface length (AISL), length of scleral spur landmark (SSL) to pupillary margin (SSL-to-PM), iris contour ratio (ICR = AISL/SSL-to-PM), pupil radius, radius of iris centroid (RICe), and iris volume were measured. Outcome variables were summarized for all eyes and age groups, and mean values among age groups were compared using one-way analysis of variance. Stepwise regression analysis was used to investigate demographic and ocular characteristic factors that affected each iris-related parameter.

**Results:**

Mean (±SD) values were 2.24 mm (±0.46), 4.06 mm (±0.27), 3.65 mm (±0.48), 4.16 mm (±0.47), 1.14 (±0.04), 1.51 mm^2^ (±0.23), and 38.42 μL (±4.91) for pupillary radius, RICe, SSL-to-PM, AISL, ICR, iris cross-sectional area, and iris volume, respectively. Both pupillary radius (*P* = 0.002) and RICe (*P* = 0.027) decreased with age, while SSL-to-PM (*P* = 0.002) and AISL increased with age (*P* = 0.001). ICR (*P* = 0.54) and iris volume (*P* = 0.49) were not affected by age.

**Conclusion:**

This study establishes reference values for iris-related parameters in an adult open angle population, which will be useful for future studies examining the role of iris changes in pathologic states.

## Introduction

Changes in iris from light to dark play an important role in the pathogenesis of primary angle closure (PAC). Quigley et al [1] and Aptel et al [2] proposed that a smaller iris cross-sectional area or iris volume change after physiologic pupil dilation may be a potential risk factor for PAC. However, accurate measurements of iris cross-sectional area and volume are difficult, since conventional imaging modalities, including ultrasound biomicroscopy (UBM) and first-generation anterior segment optical coherence tomography (ASOCT), lack the precision to accurately and reproducibly measure these parameters. Furthermore, standard reference ranges for iris volume in an open angle population have yet to be established.

Several ASOCT devices have been developed in the last decade. However, older generation ASOCT devices can image only 2 meridians at a time. Though Aptel et al recently estimated iris volume using 2 sets of ASOCT scans of 2 meridians [2], the device must be reset between scans, and the accuracy of such measurements is limited by this delay. Interestingly, Narawanaswamy et al demonstrated an increase in iris cross-sectional area and a decrease in iris volume from light to dark in patients with PAC [3]; however, ASOCT iris measurements in this study were estimated using scans at only 4 meridians. Newer generation ASOCT instruments, such as the CASIA SS-1000 (Tomey Corporation, Nagoya, Japan), utilize swept source Fourier domain technology, allowing imaging of 128 meridians (256 angles) in 2.4 seconds [4, 5], producing rapid and high resolution measurements of the entire iris.

In this study, we sought to define criteria for iris-related parameters in an adult open angle population without structural iris pathology or gonioscopically visible anterior chamber angle pathology. We evaluated the distribution of numerous iris-related parameters, including iris volume, anterior iris surface length (AISL), length of scleral spur landmark (SSL) [5] to pupillary margin (SSL-to-PM), iris contour ratio (ICR), radius of iris centroid (RICe), iris cross-sectional area, and pupillary radius, in the dark for open angle eyes using the CASIA SS-1000. Our study establishes reference values for iris-related parameters in an adult open angle population, which will be useful for future studies examining the role of iris changes in pathologic states.

## Participants and Methods

This retrospective study was conducted at the Robert Cizik Eye Clinic of the Ruiz Department of Ophthalmology and Visual Science at The University of Texas Health Science Center at Houston (UTHealth) McGovern Medical School. The University of Texas Health Science Center Committee for the Protection of Human Subjects determined that this study was exempt from review and approved the study. All research adhered to the tenets of the Declaration of Helsinki and was HIPAA compliant. Informed consent was not obtained from participants (or next of kin) for their clinical records. Patient records were anonymized and de-identified prior to analysis.

### Participants

Charts of subjects who were 18 years of age or older with open angles (open to the ciliary body band or the scleral spur in all 4 quadrants; Spaeth grades D or E [6, 7]) by gonioscopy exam and who had ASOCT images taken at the same date, between December 2012 and December 2013, were reviewed. This study population included eyes with primary open angle glaucoma (POAG, with visual field or optic disc defects), ocular hypertension (POAG suspect, due to ocular hypertension without visual field or optic disc defects), and eyes with open angles without a diagnosis of glaucoma or suspicion of glaucoma (normal). Our study population is “adult open angle” eyes and not “normal” eyes. By definition, POAG has normal appearing anterior segments, including open angles. All glaucoma patients in this study were confirmed by gonioscopy to have normal appearing angles without anterior segment abnormality.

Eyes were excluded if there was a history of intraocular surgery or laser treatment (such as lens extraction or laser peripheral iridotomy), penetrating trauma, visible structural iris or angle pathology, or any anterior segment abnormality that affected visualization of the angle or its measurements (e.g. significant corneal opacity). Participants were also excluded if they used any medication that may have affected angle anatomy within a month prior to imaging. When both eyes of the participant were eligible, 1 eye was randomly selected by coin flip. Since age is a known potential factor affecting the iris-related parameters [8, 9], participants were stratified into 5 age groups (18–35, 36–45, 46–55, 56–65, and > 66 years).

Demographics, ocular characteristics and diagnosis, including iris color, corneal abnormality, presence or absence of cataract, glaucoma diagnosis, intraocular pressure (IOP), number of IOP-lowering medications, angle grading by gonioscopic examination (using the Spaeth grading system [6, 7]) and refraction, were recorded. Age and race/ethnicity of participants was self-reported; all ocular information was based on participant self-reported histories or from chart review.

### Analysis of ASOCT Images

Imaging procedures used have been previously detailed [4]. Images were taken in a dark room (light measurement: 0 lux), and auto-alignment was engaged on the CASIA SS-1000 [4, 5]. The images were exported from the CASIA SS-1000 and read by an experienced reader using customized software, Anterior Chamber Angle and Interpretation (ACAI, Houston, TX). The reader (AZC) was masked to the gonioscopy grade. Image reading procedures have been previously described [4]. It should be noted that a previous study showed that 16 read images is sufficient to estimate iris volume [10].

**Fig 1:**
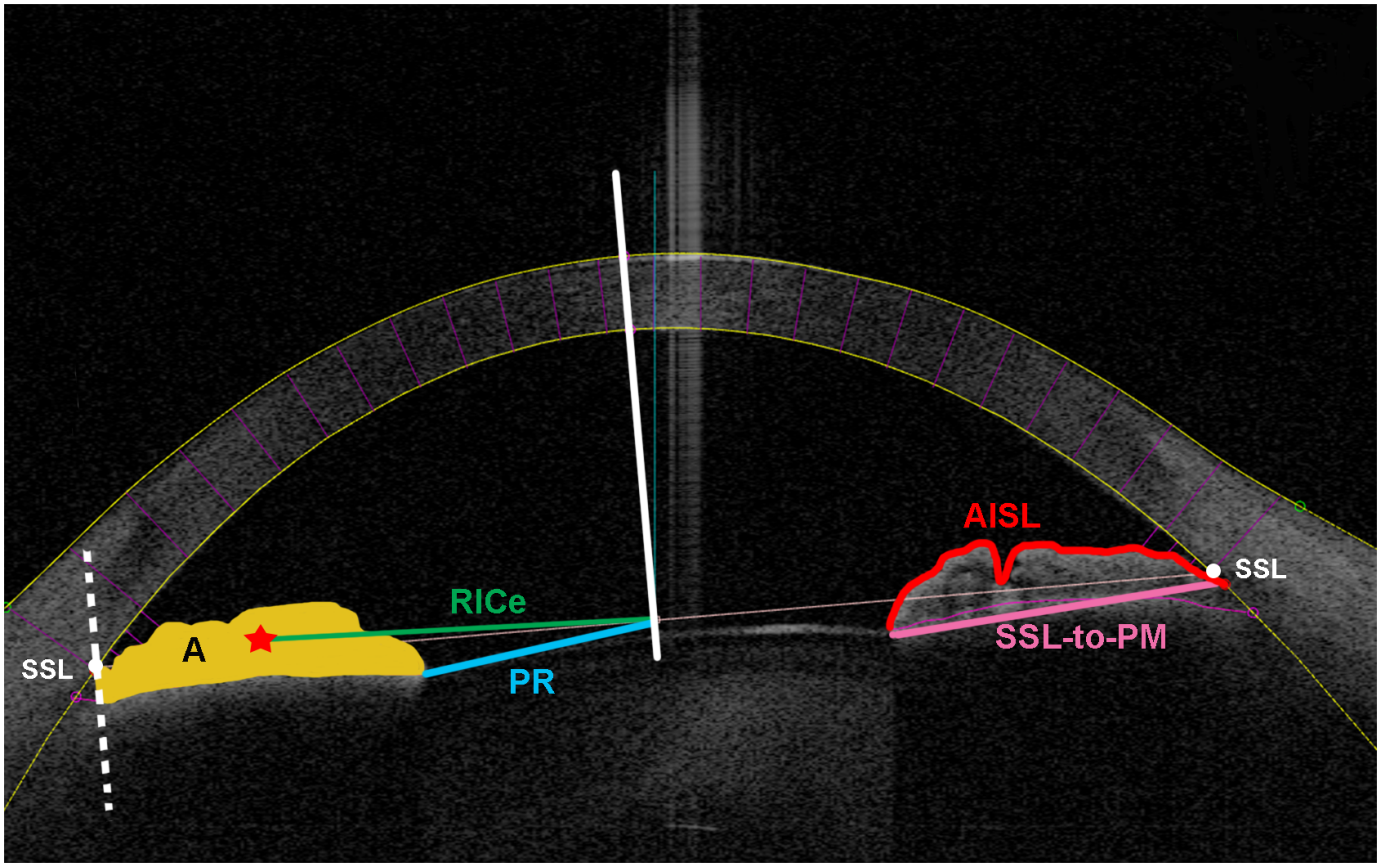
Iris-related parameters as measured by ASOCT. A = iris cross-sectional area; RICe = radius of iris centroid; SSL = scleral spur landmark; PR = pupillary radius; AISL = anterior iris surface length; SSL-to-PM = scleral spur landmark to pupillary margin.

In addition to anterior chamber angle (ACA) parameters, ACAI also enables measurements of anterior iris surface length (AISL), length of SSL to the pupillary margin (SSL-to-PM), iris contour ratio (ICR = AISL/SSL-to-PM), pupillary radius (distance from the midpoint of SSL-to-SSL line to the pupillary margin) and radius of iris centroid (RICe, distance from the midpoint of SSL-to-SSL line to the centroid of iris cross sectional area). Iris cross-sectional area (A) is defined as the area bounded by anterior iris surface, anterior surface of the demarcation line created by the reflection of the posterior layer of pigment epithelium, and a line drawn from the SSL parallel to visual axis. AISL is defined as the length of anterior iris surface from pupillary margin to the SSL (Fig 1). The iris volume was calculated using Pappus's centroid theorem formula:

$$V=2\;\pi \;\overset{32}{\underset{i=1}{\sum }}RICe_{i}\times A_{i}/32$$

### Sample Size Calculation

To establish the reference range for iris-related parameters in open angle eyes, the sample size calculation was based on the assumption that the iris-related parameters are normally distributed with constant variance among age groups. A predetermined acceptable precision for means was calculated by setting the 95% confidence interval (precision) for mean as 20% of standard deviation (SD) on either side of the estimated mean, which yielded a required sample size of 96. An approximately equal sample size (~ 20 subjects) was enrolled in each of the 5 age groups, as this was determined to be sufficient to establish the reference range with confidence width for mean as 45% of SD in each age group.

### Statistical Analysis

Demographics were summarized by mean and SD for continuous variables or by frequency (%) for discrete variables. Histograms for iris-related parameters, pupillary radius, RICe, AISL, SSL-to-PM, ICR, iris cross-sectional area, and iris volume were plotted, as well as descriptive statistics, including mean, SD, median, range, and 2.5 and 97.5 percentiles. Normality testing was performed to investigate whether the values were normally distributed. Pearson correlations among iris-related parameters were calculated. In addition, outcome variables were summarized for all eyes and each age group, and the mean values among age groups were compared using one-way analysis of variance (ANOVA). Furthermore, stepwise regression analysis was used to investigate the factors that affected each outcome variable. The factors investigated were age, gonioscopic grade (D vs E), gender, race (Black vs White vs Others), IOP, presence or absence of open angle glaucoma/suspect, presence or absence of cataract, and spherical equivalent.

All statistical analyses were performed using SAS for Windows v9.4 (SAS, Inc., Cary, NC). *P* < 0.05 was considered statistically significant for all comparisons. Data are available at https://figshare.com/s/7504c2cdda2efadfaad0, DOI: 10.6084/m9.figshare.2062893,.

## Results

A total of 98 eyes of 98 participants were included in the study. The mean (±SD) age was 50 (±15) years (range 21–79 years). There were approximately 20 participants in each of 5 age groups: 18–35, 36–45, 46–55, 56–65, and 65–79 years. Fifty-nine eyes (60%) were from female participants. Forty-two (43%) were right eyes. The study included 55 White (56%), 23 Black (23%), 10 Hispanic (10%), and 10 Asian (10%) participants. Gonioscopic findings included 57 eyes (58%) open to the ciliary body band (Spaeth grade E), and 41 eyes (42%) open to the scleral spur (Spaeth grade D). Thirteen eyes (13%) had primary open angle glaucoma (POAG) without visible structural abnormalities, and 25 eyes (26%) were POAG suspects. Two eyes (2%) had previously undergone laser-assisted in situ kertomileusis. Forty-eight eyes (51%) showed presence of cataract; in 3 eyes the presence or absence of cataract was not recorded. Ocular characteristics are given in Table 1.

**Table 1:** Ocular characteristics of 98 study eyes.

Ocular Characteristics	Statistics
	(N = 98)
Study eye, N right eye (%)	42 (43%)
Iris Color, N (%)	
Blue	22 (22%)
Brown	68 (69%)
Hazel	8 (8%)
Cornea, N (%)	
Normal	87 (89%)
Laser-assisted in situ keratomileusis	2 (2%)
Punctuate epithelial keratopathy	8 (8%)
Epithelial basement membrane dystropy	1 (1%)
Presence of cataract, N (%)*	48 (51%)
Glaucoma, N (%)^†^	
Normal	59 (61%)
POAG suspect	25 (26%)
POAG	13 (13%)
Intraocular pressure, mm Hg (±SD) ^†^	14.95 (±3.07)
Number of glaucoma medications, N (%)^†^	
0	79 (81%)
1	11 (11%)
2	6 (6%)
3	2 (2%)
Gonioscopy, N (%)	
Open to scleral spur (D)	41 (42%)
Open to ciliary body band (E)	57 (58%)
Spherical Equivalent, Diopter (±SD) ^‡^	-1.93 (±3.68)
TICV750 μL (±SD)	8.83 (±3.75)

* 3 missing data points

^†^ 1 missing data point

^‡^ 12 missing data points

TICV750 = trabecular-iris circumference volume at 750 μL from the scleral spur landmark [4]; SD = standard deviation; POAG = primary open angle glaucoma

**Fig 2:**
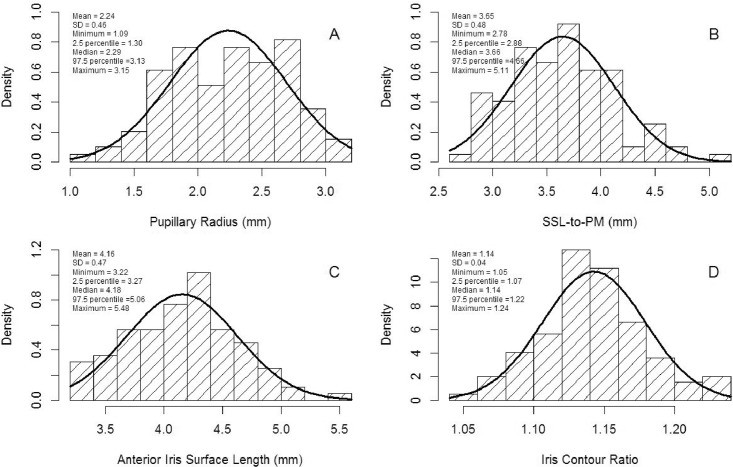
Histograms and descriptive statistics. Histograms and descriptive statistics for pupillary radius (A), scleral spur landmark to pupillary margin (SSL-to-PM; B), anterior iris surface length (C), and iris contour ratio (D).

**Fig 3:**
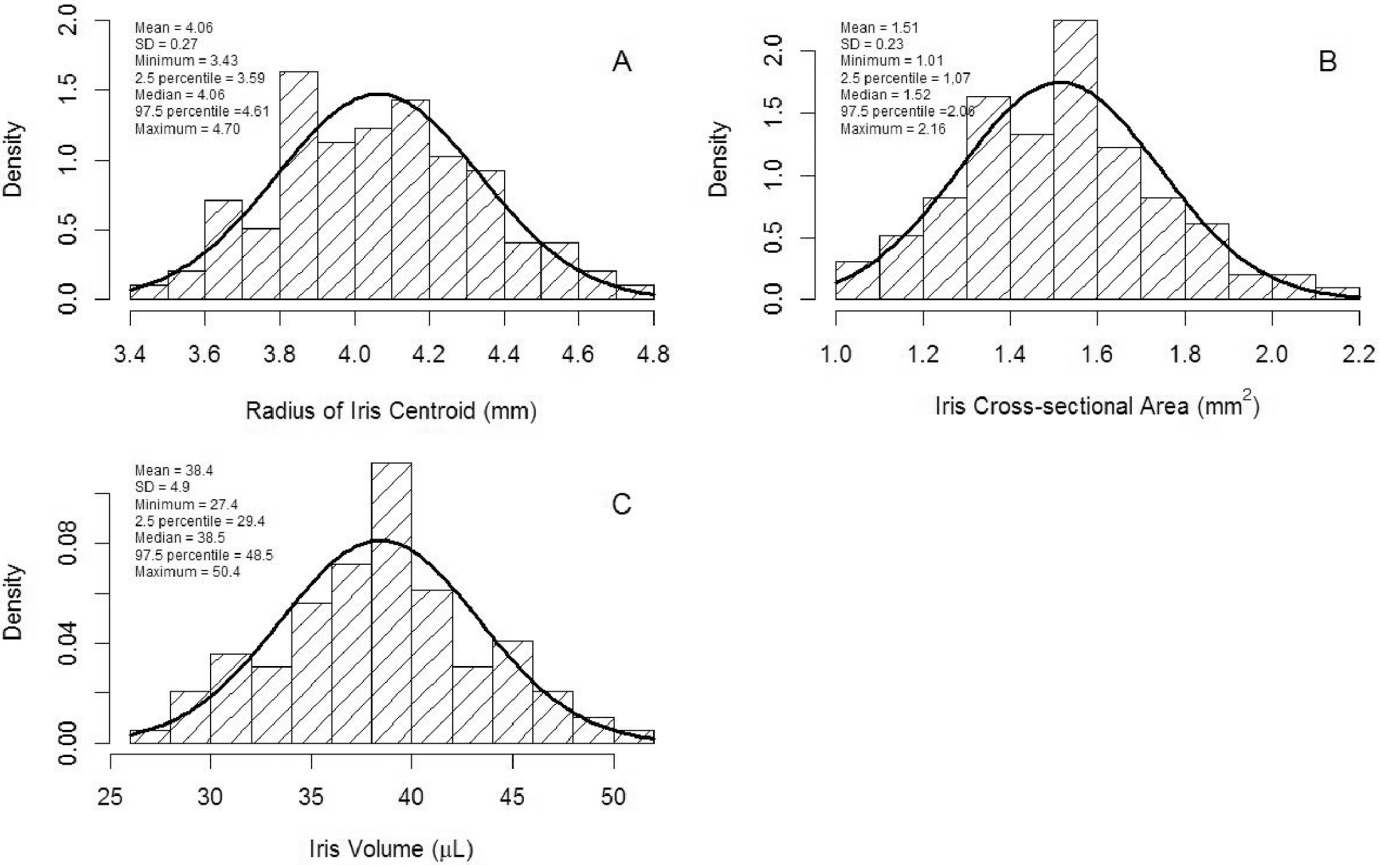
Histograms and descriptive statistics. Histograms and descriptive statistics for radius of iris centroid (A), iris cross-sectional area (B), and iris volume (C).

Histograms and summary statistics for each outcome variable are shown in Figs 2 and 3 for all eyes. The mean (±SD) values were 2.24 mm (±0.46), 3.65 mm (±0.48), 4.16 mm (±0.47), 1.14 (±0.04), 4.06 mm (±0.27), 1.51 mm^2^ (±0.23) and 38.42 μL (±4.91) for pupillary radius, SSL-to-PM, AISL, ICR, RICe, iris cross-sectional area, and iris volume, respectively. Kolomogorov-Smirnov normality testing showed that all iris-related parameters were normally distributed (*P*>0.15). Pearson correlation coefficients among iris-related parameters are presented in Table 2, which indicates that all the parameters were correlated with each other, except iris volume and RICe (R = -0.08 [*P* = 0.42]), and iris volume and ICR (R = -0.11 [*P* = 0.28]).

**Table 2:** Pearson Correlation Coefficient, R (*P* value), among Iris-Related Parameters.

Iris-Related Parameter	SSL-to-PM	AISL	ICR	RICe	Iris Cross-sectional Area	Iris Volume
Pupillary Radius	-0.92(<0.001)	-0.89(<0.001)	0.56(<0.001)	0.92(<0.001)	-0.65(<0.001)	-0.28(0.005)
SSL-to-PM		0.97(<0.001)	-0.58(<0.001)	-0.73(<0.001)	0.69(<0.001)	0.42(<0.001)
AISL			-0.38(<0.001)	-0.70(<0.001)	0.70(<0.001)	0.45(<0.001)
ICR				0.51(<0.001)	-0.30(0.003)	-0.08(0.42)
RICe					-0.54(<0.001)	-0.11(0.28)
Iris Cross-sectional Area						0.90(<0.001)

SSL-to-PM = scleral spur landmark to pupillary margin; AISL = anterior iris surface length; ICR = iris contour ratio; RICe = radius of iris centroid

### Age Effect

The means (±SD) for iris-related parameters are shown in Table 3 for all eyes and for each age group. Both pupillary radius (*P* = 0.002) and RICe (*P* = 0.027) decreased with age, while SSL-to-PM (*P* = 0.002) and AISL increased with age (*P* = 0.001). ICR (*P* = 0.54), iris cross-sectional area (*P* = 0.24) and iris volume (*P* = 0.49) were not affected by age.

**Table 3:** Outcome measurements (Mean [±SD]) for all eyes and for eyes in each age group.

Iris-Related Parameter	Age (years)						
	All (N = 98)	≤ 35 (N = 19)	36–45 (N = 21)	46–55 (N = 20)	56–65 (N = 19)	>65 (N = 19)	*P* Value
Pupillary Radius, mm	2.24 (±0.46)	2.50 (±0.47)	2.35 (±0.41)	2.25 (±0.44)	2.13 (±0.36)	1.95 (±0.44)	0.002
SSL-to-PM, mm	3.65 (±0.48)	3.37 (±0.51)	3.55 (±0.43)	3.62 (±0.44)	3.74 (±0.47)	3.96 (±0.36)	0.002
AISL, mm	4.16 (±0.47)	3.87 (±0.52)	4.05 (±0.41)	4.14 (±0.47)	4.24 (±0.43)	4.49 (±0.32)	0.001
ICR	1.14 (±0.04)	1.15 (±0.03)	1.14 (±0.04)	1.15 (±0.04)	1.14 (±0.04)	1.13 (±0.04)	0.54
RICe, mm	4.06 (±0.27)	4.19 (±0.29)	4.14 (±0.26)	4.06 (±0.26)	3.99 (±0.20)	3.94 (±0.29)	0.027
Iris Cross-Sectional Area, mm^2^	1.51 (±0.23)	1.43 (±0.29)	1.52 (±0.23)	1.53 (±0.18)	1.49 (±0.22)	1.60 (±0.20)	0.24
Iris Volume, μL	38.42 (±4.91)	37.31 (±5.99)	39.26 (±5.39)	38.72 (±3.58)	37.29 (±5.37)	39.41 (±3.85)	0.49

SSL-to-PM = scleral spur landmark to pupillary margin; AISL = anterior iris surface length; ICR = iris contour ratio; RICe = radius of iris centroid

### Causative Factors Affecting Iris-related Parameters

Table 4 shows the factors identified that were significantly correlated with iris-related parameters and estimates of the magnitude of the effects from stepwise regression analysis for each iris-related parameter. The age effect was consistent with the findings obtained from one-way ANOVA. Pupillary radius was estimated to be 0.30 mm (±0.10) shorter, SSL-to-PM was 0.23 mm (±0.11) longer, AISL was 0.26 mm (±0.11) longer, iris cross-sectional area was 0.22 mm^2^ (±0.05) larger, and iris volume was 3.30 μL (±1.18) larger in Black than other races. ICR was 0.02 (±0.01) higher in Whites than other races. On average, iris cross-sectional area and iris volume in male participants was 0.10 mm^2^ (±0.05) and 2.78 μL (±1.00), respectively, larger than in females.

**Table 4:** Estimated Regression Coefficient (β) (±standard error) as well as associated *P* value.

Significant Factor	Pupillary Radius (mm)	SSL-to-PM (mm)	AISL (mm)	ICR	RICe (mm)	Iris Cross-Sectional Area (mm^2^)	Iris Volume (μL)
Age (per 10 years)	-0.11 (±0.03) [*P*<0.001]	0.12 (±0.03) [*P*<0.001]	0.10 (±0.03) [*P* = 0.004]		-0.04 (±0.02) [*P* = 0.018]		
Sex (Male vs Female)						0.10 (±0.05) [P = 0.03]	2.78 (±1.00) [P = 0.007]
Race (Black vs others)	-0.30 (±0.10) [*P* = 0.004]	0.23 (±0.11) [*P* = 0.04]	0.26 (±0.11) [*P* = 0.024]			0.22 (±0.05) [*P* = <0.001]	3.30 (±1.18) [*P* = 0.006]
Race (White vs others)				0.02 (±0.01) [*P* = 0.028]			
POAG (vs others)				-0.02 (±0.01) [*P* = 0.008]			
Spherical Equivalent, D			0.03 (±0.01) [*P* = 0.037]				
Gonioscopic grade (Open to ciliary body band [E] vs to scleral spur [D])					0.18 (±0.06) [*P* = 0.002]		

SSL-to-PM = scleral spur landmark to pupillary margin; AISL = anterior iris surface length; ICR = iris contour ratio; RICe = radius of iris centroid; D = diopter; POAG = primary open angle glaucoma

Of ocular characteristics documented, primary open angle glaucoma (POAG), spherical equivalent, and iris insertion were found to significantly affect iris-related parameters. ICR decreased in eyes with POAG by 0.02 (±0.01). AISL was estimated to increase 0.03 mm (±0.01) with each +1.00 diopter increase in spherical equivalent. Iris insertion examined by gonioscopy was correlated with RICe; deeper angles were associated with a greater RICe.

## Discussion

In this study we have established reference values for a variety of iris-related parameters in an adult open angle population, as measured by swept source Fourier domain ASOCT. Previous studies have examined iris-related parameters [11–13], including iris area and iris length in open angle populations, but have been limited by technologies such as UBM and first-generation ASOCT that yield less accurate and reproducible measures [14, 15]. Using the CASIA SS-1000, we obtained high resolution images of the entire iris and then evaluated the distribution of iris-related parameters in the dark in eyes with open angles.

The ACAI software enables the measurement of a number of novel iris-related parameters that allow for detailed characterization of the iris, including AISL (the length of the anterior surface of the iris stroma), SSL-to-PM (the length of the posterior surface of the iris stroma), and their ratio (ICR). Factors that would be expected to increase the ICR include bowing, edema, and a “bumpy” surface of the iris. The ICR of a flat, more compact iris should have a value closer to 1.0. As expected, we found that pupillary radius and RICe decreased with age, while SSL-to-PM and AISL increased with age. It has been well established that pupil size decreases with age [16], and our findings reflect this. ICR did not change with age, suggesting that the increases in both SSL-to-PM and AISL with age are relatively proportional.

We found that iris volume in our study population was not affected by age, similar to a recent study of a White population by Invernizzi et al [8]. This finding is in agreement with a recent study by Tun et al [17] demonstrating that iris volume measured with Fourier domain ASOCT in a Chinese population had a similar mean value (ours = 38.42 ± 4.91 μL, Tun’s = 36.70 ± 4.1 μL) and did not change with age. The clinical significance of this is yet to be determined. It is also important to note that in both studies iris-related parameters were measured in the dark only. Future investigations will be needed to determine if dynamic changes in iris volume (i.e. from light to dark) are affected by age.

Interestingly, a number of recent studies using ASOCT have suggested that structural and dynamic changes in iris cross-sectional area and volume are likely to play a role in the development of PAC [3, 14, 17, 18]. Zhang et al found differential iris responses between PACS and open angle eyes to physiologic or pharmacologic dilation [14], while Tun et al demonstrated that iris volume is greater in closed angles compared to open angles [17]. Because these studies were limited to Chinese populations, the relevance of these findings to other ethnicities in the development of PAC, or to the development of other iris-related disorders, is uncertain, especially given the recent study by Seager et al demonstrating unique iris-related parameters, especially iris area, in a Chinese population [18]. Of note, the pupil diameter reported by Zhang et al (4.78 ± 0.64 mm) [14] correlates with our measures of pupillary radius (2.23 ± 0.46 mm). However, their measure of open angle iris volume in the dark (28.53 ± 3.21 μL) is markedly different from ours (38.42 ± 4.91 μL), which also differs considerably from that of Narayanaswamy et al (42.33±7.97 μL) [3]. Both Zhang et al [14] and Naravanaswamy et al [3] measured iris volume predominately in Chinese participants, using the same software (ZAAP) and first-generation ASOCT (Vistante, Carl Zeiss Meditec, Dublin, CA). Invernizzi et al measured iris volumes smaller than ours in a White population with various iris colors, also using the same CASIA SS-1000 [8] technology used in our study. While it is unclear as to why iris volume varied between these studies, the dissimilarity of our measurement to both is likely related to population and image acquisition differences, as well as variability in criteria for outlining the iris border. The same differences limit comparison of our iris cross-sectional area measurements to other studies [3, 14, 18].

In our study we have also identified a number of relationships between clinical characteristics and iris-related parameters that will be useful in future studies of the role of iris changes in pathologic states. Iris volume was larger in males than in females; AISL increased with spherical equivalent, which makes sense considering that high hyperopes are more likely to have relative pupillary block with iris bowing; and RICe correlated with deeper angles measured by gonioscopy. With regard to race, ICR was higher in Whites; while in Black participants, pupillary radius was shorter and AISL was longer, suggesting a relative miosis in this population. While the clinical significance of these findings remains to be seen, it will be important to adjust for these relationships in future studies of iris-related parameters in different populations. Interestingly, we also found that iris volume was larger in Black participants. Given the higher rate of open angle glaucoma in the Black population [19, 20], it is intriguing to speculate on the possible role of iris volume in the pathogenesis of this disease, and further studies are needed to establish the role of iris volume in the development of open angle glaucoma.

Further studies are also needed to establish the clinical utility of measuring the anterior surface of the iris (AISL), the straight line distance between the scleral spur and pupillary margin (SSL-to-PM), and the ratio of their lengths (ICR). We anticipate that these parameters are related to the amount of anterior bowing of the iris. A bowed or edematous iris would be expected to have a greater AISL relative to SSL-to-PM and would thereby have a higher ICR. Interestingly, ICR was relatively lower in eyes with open angle glaucoma than those without a diagnosis of POAG, though the clinical relevance of this remains to be determined. One could speculate that chronic use of IOP-lowering medications may result in atrophic structural changes in the iris stroma, but this study was not designed to investigate this question. Conversely, although not specifically evaluated in this study, one might expect higher ICR values in eyes with primary angle closure (PAC) due to relative anterior iris bowing with mild iris stromal edema. With successful treatment of PAC by laser iridotomy or lens extraction, a decrease in ICR should be seen as the iris bowing and pupillary block is relieved.

A limitation of this study is related to the inability of ASOCT to image through heavily pigmented structures such as the iris pigment epithelium (IPE). As a result, the volume of the entire iris cannot be accurately measured because the posterior surface of the IPE cannot be delineated. Therefore, the demarcation line between the posterior surface of the iris stroma and the more heavily pigmented IPE was used when calculating the iris cross-sectional area and volume. Structural variations in the IPE among the cohorts studied may be difficult to detect. One also must be cautious when comparing iris volumes between studies using different analytical software because of differences in how the iris borders are defined.

Another limitation is that we only studied eyes with open anterior chamber angles. Although the population was racially heterogenous, the range of iris and anterior segment anatomic variability was limited. Now that open angle reference values have been established, future studies could compare with eyes on the primary angle closure spectrum, since their anatomy is typically distinct from eyes with open angles. This will permit better observation of the how iris-related parameters may be impacted by variations in biometric properties such as axial length, keratometry, lens thickness, and anterior chamber depth.

Although the sample size may appear small, the sample size of 20 in each of the 5 age groups is sufficient to establish references values with the confidence interval width < 4% of mean for open angle eyes and within 9% of mean in each age group. We believe the precision of the reference values was satisfactory. Many studies in the literature have investigated “normative” measurements with a sample size of approximately 100 [21–23]. While we have sufficient power to identify causative factors (such as those described in Table 4), we do not have sufficient precision to adjust the absolute reference values we were able to discern in this study based on the causative factors identified. However, identification of these factors would not have occurred except in a study designed as this one. The goal of the study, to establish reference values for iris-related parameters in an open angle population, was met with our sample size.

In summary, we have established reference values for iris-related parameters in an adult open angle population. These values will be useful for future studies examining the role of iris changes in the pathogenesis of both open and closed angle glaucoma.
